# Management of Hypnotics in Patients with Insomnia and Heart Failure during Hospitalization: A Systematic Review

**DOI:** 10.3390/nursrep11020036

**Published:** 2021-05-21

**Authors:** Pablo Jorge-Samitier, María Teresa Fernández-Rodrigo, Raúl Juárez-Vela, Isabel Antón-Solanas, Vicente Gea-Caballero

**Affiliations:** 1 Hospital Clínico Lozano Blesa, Avenida San Juan Bosco, 15, 50009 Zaragoza, Spain; pjorge@salud.aragon.es; 2 Department of Physiatry and Nursing, Faculty of Health Sciences, University of Zaragoza, C/Domingo Miral s/n, 50009 Zaragoza, Spain; 3 Faculty of Medicine, University of Salamanca, Avenida Alfonso X El Sabio SN, 37008 Salamanca, Spain; raul.juarez@unirioja.es; 4 Research Group GENIAPA (GIIS094), Institute of Research of Aragon, Avenida San Juan Bosco, 13, 50009 Zaragoza, Spain; 5 Nursing School La Fe, Adscript Center of the University of Valencia, Research Group GREIACC, Health Research Institute La Fe, 46026 Valencia, Spain; gea_vic@gva.es

**Keywords:** heart failure, insomnia, sleep disorder, hypnotic, benzodiazepine, elderly

## Abstract

Background: Heart failure is a chronic, progressive syndrome of signs and symptoms, which has been associated to a range of comorbidities including insomnia. Acute decompensation of heart failure frequently leads to hospital admission. During hospital admission, long-term pharmacological treatments such as hypnotics can be modified or stopped. Aim: To synthesize the scientific evidence available about the effect of withdrawing hypnotic drugs during hospital admission in patients with decompensated heart failure and insomnia. Method: A systematic review of the literature following the Preferred Reporting Items for Systematic reviews and Meta-Analyses (PRISMA) guidelines was carried out in the following scientific databases: PubMed, Scopus, Dialnet and Cochrane. Inclusion criteria: studies including a population of adults with heart failure and sleep disorders in treatment with hypnotics and admitted to hospital, studies written in English or Spanish and published until June 2020. Exclusion criteria: studies involving children, patients admitted to intensive care and patients diagnosed with sleep apnea. Results: We identified a total of 265 documents; only nine papers met the selection criteria. The most frequently used drugs for the treatment of insomnia in patients with heart failure were benzodiazepines and benzodiazepine agonists; their secondary effects can alter perceived quality of life and increase the risk of adverse effects. Withdrawal of these drugs during hospital admission could increase the risk of delirium. Future research in this area should evaluate the management of hypnotics during hospital admission in patients with decompensated heart failure. In addition, safe and efficient non-pharmacological alternatives for the treatment of insomnia in this population should be tested and implemented.

## 1. Introduction

Heart failure (HF) is a chronic, progressive syndrome of signs and symptoms in which the heart is not able to meet the metabolic demands of the body or does so at the cost of increasing ventricular filling pressure [1]. HF is one of the greatest public health problems worldwide due to its complex and progressive nature, loss of quality of life, frequency of hospital admissions and high rate of mortality [2,3,4]. In Spain, HF affects 6.8% of the population and its incidence increases with age (8% of HF patients are aged 65–75 and 16.1% are over 75) [3,5].

HF is frequently associated with sleep disorders (SD) such as insomnia (over 75% of patients with HF experience SD) [2,6,7]. SD are defined as disorders whose symptoms or pathophysiology are related with sleep regardless of comorbid physical and/or mental disorders. Patients with SD have difficulty falling or maintaining sleep and experience excessive daytime sleepiness [8,9]. Frequent manifestations of SD include orthopnea, nycturia and restless legs syndrome, and it is associated with older age and worst survival rates [10,11]. In patients with HF, SD have a negative impact on physical health, cognitive efficiency, daily activity, mental health and disease progression [10,11]. Specifically, patients with HF and SD have a reduced ability to face new problems [12], stay alert and remember new things, which results in poor treatment adherence and self-care problems [13,14,15,16,17,18]. 

HF patients who experience insomnia are frequently treated with hypnotic medication in order to palliate the symptoms outlined above. In fact, between 9.5% and 30% of patients with HF take hypnotics regularly [11,12,16,19]. In Spain, this percentage increases significantly, with 82.4% of HF patients having taken hypnotics before, and 35% having taken them occasionally, frequently or continuously in the week before data collection, according to a recent study [20]. Unfortunately, frequent secondary effects of hypnotic drugs include respiratory depression, tolerance and dependency [21].

Acute decompensated HF can be defined as the sudden or gradual onset of the signs or symptoms of HF requiring unplanned use of health services, including hospitalization [6]. This results in increased healthcare expenditure (1.8–3.1% of the total health public budget) [3,5], especially in older adults [1], with an average length of hospital stay of 9 +/− 5 days [5]. During hospital admission, the pharmacological treatment of HF patients with and without insomnia is often modified, which could cause complications if not carefully monitored. Specifically, an incorrect approach to insomnia in patients admitted to hospital with decompensated HF can cause complications in the short, medium and long term [9]. Thus, Aim: this paper aims to analyze and assess the evidence available on the management of hypnotics and the consequences of sedative–hypnotic drug withdrawal during hospital admission in patients with a primary diagnosis of HF and insomnia.

## 2. Material and Methods

### 2.1. Design

We conducted a systematic review of the literature in order to synthesize the evidence about the use (and withdrawal) of hypnotics in patients admitted to hospital with HF and insomnia. This report followed the PRISMA guidelines [22]. The PICO question (Ask, Intervention, Comparation, Outcomes) comprised older adults with insomnia admitted to hospital with decompensated HF (Population), in treatment with hypnotics (Intervention), managed during hospital admission (Comparison), evaluating the consequences of hypnotic withdrawal as well as the application of alternative treatments for insomnia (Outcome). In short: does adequate management of hypnotics in older patients with insomnia and HF help to prevent complications during hospital admission?

### 2.2. Search Strategy

We used the Preferred Reporting Items for Systematic Reviews and Meta-Analyses (PRISMA) guideline for the identification and selection of studies [22]. 

The following terms were included in the search formulae and were combined with the Boolean operators AND or OR as appropriate: heart failure, hypnotics, sedatives, benzodiazepines (Bz) and insomnia (Table 1). No additional filters were used in order to avoid losing any relevant articles. The search was completed between May and June 2020. A total of 265 preliminary results were screened in order to identify those which were irrelevant and/or did not meet the selection criteria. The remaining articles were examined in depth in the second round according to the PRISMA guidelines.

### 2.3. Selection Criteria

Inclusion criteria: studies including adults with HF and insomnia in treatment with hypnotics and admitted to hospital, studies written in English or Spanish and published on any date. Exclusion criteria: grey literature, review and commentary articles, qualitative studies and conference papers; studies including children; studies including adults admitted to intensive care and patients diagnosed with sleep apnea in treatment with mechanical ventilation. We excluded these last 2 groups due to significant differences between both patient populations in terms of their health status, the characteristics of the unit, and the specific therapies used in the treatment of their respiratory condition.

### 2.4. Data Extraction and Synthesis

**Figure 1:**
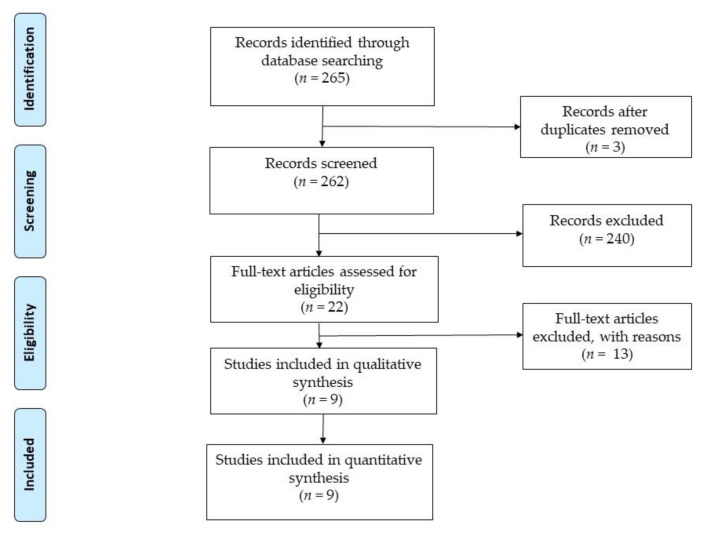
Flow char.

The search strategy produced a total of 265 potentially relevant studies: 134 in PubMed, 89 in SCOPUS, 36 in Cochrane and 9 in Dialnet. Two researchers carried out the search separately. We reviewed the titles and abstracts of a total of 265 articles in order to identify any duplicate and/or unrelated documents; 243 duplicate and/or unrelated documents were excluded after reading the title and abstract. Subsequently, 2 researchers independently reviewed the remaining 22 full-text articles and 13 more were excluded due to not meeting the selection criteria. Finally, 9 articles were found eligible and were included in this systematic review. The selection process can be seen in Figure 1.

### 2.5. Quality Assessment

The Quality Assessment Tool for Quantitative Studies (QATQS) described by the Effective Public Healthcare Panacea Project (EPHPP) [23]) was used to appraise the quality of the articles identified, expressed in Table 2. Subsequently, all the articles were appraised using the PRISMA 2009 checklist. After careful consideration of the results from the quality appraisal methods, we decided to include all 9 articles in this review [24]. 

## 3. Results

The nine studies comprised in this systematic review were published between 2007 and 2018 (Table 3). Five were narrative or systematic reviews, two were descriptive, one was correlational and one was experimental. The sample size ranged between 15 and 22,684 participants.

All the papers included in this systematic review addressed the effects of hypnotics and other sedatives on patients with HF [25,26,27,28,29,30,31,32,33]. In their systematic reviews, Motter et al. [25] and Ishak et al. [26] offer an exhaustive description of the use hypnotics, as well as their side effects and interactions, and describe other therapies aimed to improve the quality of sleep, in patients with HF.

The four analytic studies included in this review evaluated the presence of insomnia and/or the severity of its symptoms using different methods of assessment. Buxó et al. [27] used the Insomnia Severity Index to classify insomnia into conciliation insomnia, maintenance insomnia and early awakening insomnia; Gatti et al. [30] used the Pittsburg Sleep Quality Index (PSQI) in order to assess sleep quality in their sample; Chung et al. [31] and Garrido et al. [29] analyzed the onset of indirect insomnia (and other sleep disturbances) after treatment with hypnotics.

Buxó et al. [27] highlighted that 48.5% of their sample presented insomnia; an association was found between insomnia and higher New York Heart Association (NYHA) functional class. In addition, patients with HF who presented insomnia had a higher incidence of adverse effects, including cardiovascular and respiratory complications frequent hospital admission and death [25,29,31,33]. Specifically, according to Buxó et al. [27], patients with HF and sleep disturbances had a higher incidence of hospitalization and death during follow-up (21% as opposed to 0% *p* < 5). 

Among other insomnia-related adverse effects, Chung et al. [31] described coughing, excessive mucus production, dyspnea and nocturnal oxygen desaturation. In addition, older adults with chronic respiratory problems often presented comorbidities including HF, sleep disturbances, depression, anxiety and dementia. These authors [31] suggest that older adults in treatment with Bz had a 1.45-fold risk of respiratory exacerbation and a 1.92-fold risk of attending an accident and emergency service, and conclude that use of Bz and other hypnotic drugs is associated with respiratory adverse events including pneumonia, Chronic Obstructive Pulmonary Disease (COPD) exacerbation, acute respiratory failure and cardiac failure.

Barbiturates were amply used throughout the 20th century for the treatment of insomnia despite their potentially harmful secondary effects as continued use causes both tolerance and dependence [28]. Subsequently, Bz have become one the pharmacological treatments of choice for dealing with insomnia, displacing barbiturates as the treatment of choice because of their fewer side effects, especially those related to dependence and tolerance [25,26,28,32,33]. However, Bz are not exempt from problems. Bz are potentially dangerous drugs, especially in the elderly [25,28,29], and can generate problems in terms of drug interactions and side effects, including accidental falls, fractures, cognitive impairment [25,26], decreased mental acuity and motor skills, daytime sleepiness [28,32,33], dependency and delirium [25,26,32,33]. Specifically, a systemic review from Motter et al. [25] suggested that Bz were inappropriate for the treatment of incoming in older adults with HF, patients with COPD and sleep apnea due to their frequent interactions and respiratory depressant effect [33]. Furthermore, discontinuing Bz may cause withdrawal symptoms or relapse. Unfortunately, long-term use of Bz is still frequent in older adults, ranging from 12 to 43% [25].

In recent years, new, safer pharmacological therapies have emerged, including Bz receptor agonists such as zolpidem, zaleplon and eszoplicone [30,32,33], which appear to have fewer side effects and improve the quality of sleep in the population of older adults with HF [33]. Specifically, according to Gatti et al. [30], Bz receptor agonists produced fewer side effects, increased total sleep time, improved the efficiency of sleep, did not increase the rate of apnea and hypopnea and decreased low saturation measures without affecting respiratory effects of central origin. However, some authors [30,33] maintain that there remains a risk of adverse respiratory events, dependence and abuse in patients with HF.

The use of pharmacotherapy to address insomnia in older adults with HF can be hazardous. Therefore, it is important that treatment with Bz and Bz receptor agonists is closely monitored in the long term [26,28,29,30,31,33]. In an attempt to avoid some of the problems related to the pharmacological treatment of insomnia in patients with HF, other non-pharmacological treatments have emerged, including sleep hygiene (multiple habits that promote good sleep), relaxation techniques (muscle relaxation, meditation and biofeedback to reduce arousal), stimulus control therapy (techniques designed to re-establish the bedroom as a signal to sleep), sleep restriction therapy (treatment that improves sleep efficiency by restricting total time in bed to number of hours the patient usually sleeps and gradually, time allowed for sleep is increased) and cognitive behavior therapy (CBT) (CBT is a catchall term combining all the different forms of non-pharmacologic treatments, which consist of changing faulty beliefs and attitudes about sleep) [31,33]. These non-pharmacological interventions do not produce immediate results and require intense and continuous training, but they do have good results in the medium to long term and should, therefore, be considered in clinical practice [26,27,32,33]. The recent National Institutes of Health (NIH) conference consensus statement on insomnia noted that the evidence supports the efficacy of CBT in the treatment of insomnia, at least in the short term. Additionally, the American Academy of Sleep Medicine (AASM) has endorsed multicomponent CBT as an effective treatment for chronic insomnia [33].

## 4. Discussion

There is a clear association between HF and SD [6,7,8,9,10,11,12,13,14,15,16,17,18], as can be observed in the articles analyzed [25,26,27,28,29,30,31,32,33]. According to Buxó et al. [27], 42% of their participants experienced conciliation insomnia, 79% maintenance insomnia and 54% early awakening. Specifically, maintenance insomnia was associated with increased morbidity and mortality, resulting in an increase in healthcare expenditure due to hospital readmission and prolonged hospital stay [3,5]. Sedative–hypnotics, specifically Bz, have been “the gold standard” for the treatment of not only insomnia [25,26,27,28,29,30,31,32,33], but also agitation, delirium [29], anxiety and depression [27] in older adults. Specifically, long-lived Bz such as diazepam and clonazepam and intermediate-lived Bz such as lorazepam or alprazolam [25,29,32] have frequently been used in the population of older adults with HF and insomnia.

Older adults are more vulnerable to Bz-related adverse effects because of physiological changes produced by aging. In particular, these physiological changes alter the pharmacokinetics and pharmacodynamics of Bz in the body, slowing down the elimination of metabolites and prolonging the action of the molecules, which may increase the risk of residual effects during the day [28,29,33]. In addition, many older adults present comorbidities, thereby increasing the number and type of drugs consumed and, subsequently, increasing the risk of drug interactions. Frequent adverse events in the population of older adults on treatment with Bz include accidental falls and delirium, which are associated with higher rates of hospitalization and mortality [25,26,28,29,33], and generate extraordinary costs to the health system [25,33]. In fact, Bz are potentially inappropriate for consumption by the elderly [25,29], especially diazepam [26].

The use of pharmacotherapy to induce or maintain sleep is significantly associated with deterioration of perceived quality of life in the general population mainly due to its side effects, namely respiratory depression, daytime sleepiness, and cognitive impairment [25,26,28,29,33]. In the population of older adults, Bz use has been correlated with serious drug-related adverse events including impaired cognitive function, delirium, accidental falls and fractures, respiratory problems including pneumonia and acute respiratory failure and cardiorespiratory arrest [25,28,29,32]. This results in increased hospital readmissions, morbidity and mortality, and represents a heavy burden to health services worldwide [25,26,27,28]. Nevertheless, some advantages have been cited in the use of Bz in the population of patients with HF and insomnia. Specifically, when adequate doses are used, Bz do not produce severe respiratory depression and present a lower risk of abuse and dependence than barbiturates [28]. However, even when appropriate doses are prescribed, the risks of dependence, tolerance and insomnia of rebound persist in the long term [25,26,28,29,32,33]. 

In the context of decompensated HF, patients frequently experience dyspnea and require acute hospital admission. After admission, chronic pharmacological treatments are often modified in order to adapt to the patient’s needs and respond to the evolving situation. For example, sedative–hypnotic drugs may be withdrawn in order to improve the patient’s respiratory function. However, there may be complications following hypnotic withdrawal in the short term [9]. Bz withdrawal symptoms are characterized by anxiety, insomnia, hypertension and tachycardia among others [25,26,28,29,33]. Patients with HF and insomnia should be closely monitored when these drugs are modified or stopped during hospital admission [25,26,32,33].

New pharmacological treatments, including Bz receptor agonists, may be a better alternative for the treatment of insomnia in the older adults with HF [26,28,30,32,33]. This group of drugs seems to have a better sedative effect during all phases of sleep, improves sleep quality, both physiological and perceived, and improves the evolution of apneas and hypopneas [30]. In addition, they are effective to improve sleep quality, increase total sleep time and prevent early awakening in the population of older adults [33], and present fewer and less severe side effects including less tolerance and dependence [28,30]. Furthermore, different studies have supported the possibility of using cognitive therapy to improve the quality of sleep and insomnia in this population. However, it is important to bear in mind that the results from cognitive therapy are not immediate and require effort and training [26,27,32,33].

## 5. Conclusions

The pharmacological management of insomnia may generate problems in the population of older adults with HF. Sedative hypnotics can cause respiratory problems, including respiratory failure and, for this reason, treatment with Bz may be paused during hospitalization in patients admitted with decompensated HF. However hypnotic withdrawal can cause anxiety and cardiovascular symptoms that can complicate patient outcomes like delirium during hospital admission. Treatment with Bz should be closely monitored in older patients with HF and insomnia. 

More studies are needed in order to analyze the effect of hypnotic withdrawal in older adults with HF and insomnia admitted to hospital with decompensated HF on disease progression, morbidity and mortality, especially in terms of the effect of hypnotic drug withdrawal (anxiety, rebound insomnia, tachycardia, restlessness) on the risk of delirium. In addition, alternative pharmacological and non-pharmacological therapies should be considered in the treatment of older adults with HF and SD. A combination of short-term pharmacological therapies and cognitive therapy training may be a suitable alternative and should be compared with existing, riskier pharmacological treatments, such as Bz.

## 6. Limitations

The evidence currently available on the research question, that is, the management of hypnotics and the consequences of sedative-hypnotic drug withdrawal during hospital admission in patients with a primary diagnosis of HF and insomnia, is scarce. This had an impact on the number, and quality, of the studies that met the selection criteria. Thus, more high-quality research on this topic will be needed in order to extract solid, evidence-based conclusions which can guide practice and future research.

It would have been preferrable to undertake a metanalysis of the findings from previous studies. However, this option was discarded due to the heterogeneity of the methodological designs, objectives and sample sizes.

## Figures and Tables

**Table 1:** Search strategy and formulae.

Database	Search Formula
Pubmed	(“Heart Failure”[Mesh]) AND (“Hypnotics and Sedatives”[Mesh] OR benzodiazepine)
Cochrane	“heart failure” AND hypnotics AND benzodiazepine
Scopus	“heart failure” AND hypnotics AND benzodiazepine
Dialnet	Insuficiencia cardiaca (insomnio OR hipnótico)
Total	265

**Table 2:** EPHPP scale. Quality Assessment tool for quantitative studes.

EPHPP	A Selection Bias	B Study Design	C Confounders	D Blinding	D Data Collection Method	E Withdrawals and Dropouts	GLOBAL RATING
**Motter**	1	1	1	1	1	1	Weak
**Garrido**	2	3	3	1	2	1	Weak
**Ishak**	1	1	1	1	1	1	Weak
**Buxó**	2	3	3	1	1	1	Weak
**De Pablos**	1	1	1	1	1	1	Weak
**Gatti**	1	1	1	1	1	1	Strong
**Chung**	1	2	1	3	1	1	Moderate
**Hayes**	1	1	1	1	1	1	Weak
**Nichols**	1	1	1	1	1	1	Weak

**Table 3:** Structed summary of results.

Author and Year	Aim	Study Design	Sample	Studied Drug	Major Side Effect	Studied Risk	Recommendation
Nichols et al., 2007	Insomnia management strategies description	Narrative review		Cognitive therapy Antihistamines Anti-depressants Bz* AgoBz** Melatonin agonist receptor	Anticholinergic effects in the elderly Sedation Daytime sleepiness Tolerance Dependence	Cardiovascular complications Adverse Breathing Events Delirium Abuse	Cognitive therapy AgoBz**
Hayes et al., 2009	Review of the effects of medication on SD in HF patients	Narrative review		Medications for the management of HF Medication for the management of insomina Cognitive therapy	Tolerance, dependence and rebound insomnia	Daytime sleepiness	AgoBz** have fewer adverse events than Bz*
De Pablos et al., 2009	Description of therapies used in patients >65 with respiratory and cardiac insufficiencies with insomnia and/or anxiety	Narrative review		Bz* AgoBz** Serotonergic Anxiolytics Antihistamines Clomethiazole	Sleepiness, decreased mental acuity and motor skills	Dependence and withdrawal in short lived Bz	Lorazepam, zolpidem, clomethiazole
Ishak et al., 2012	Study of the impact of insomnia on the quality of life	Systematic review	58	Bz* Non-pharmacological treatment	Psychiatric or physical comorbidity. Increased medication or psychosocial problem	Long-term dependence and tolerance. Deteriorating quality of life. Increased morbidity and mortality	AgoBz** to address insomnia.
Garrido et al., 2014	Study of inappropriate use of Bz* in >65	Descriptive	222	Psychotropics Bz*	Dependence, cognitive impairment, ataxia, syncope, falls, delirium and readmission to hospital	Use Bz* and AgoBz**	Reduce Bz* prescription Strict control of Bz* treatment in the long term
Chung et al., 2015	To assess the effects of hypnotics on the risk of adverse events in patients with cardiopulmonary problems	Retrospective analytical case control study	22.684	Bz* and no Bz*	Pneumonia Acute Exacerbation Acute respiratory failure Cardiorespiratory arrest	High risk of adverse respiratory events	Medical management of hypnotic treatment of patients with cardiorespiratory problems
Gatti et al., 2016	To assess the effects of zolpidem in patients with HF and sleep disorder	Double-blind, randomized, placebo-controlled analysis	15	Zolpidem	No adverse effects	Possible adverse effects	No adverse effects using Zolpidem
Buxo et al. 2018	Prevalence of insomnia in HF patients studied	Descriptive	68				Non-pharmacological measures
Motter et al., 2018	Study of potentially inappropriate drugs in >65	Systematic review	36	Bz*	Cognitive impairment, Respiratory failure	Falls, fractures, delirium	Avoid using Bz*

Bz* = Benzodiazepine; AgoBz** = Bz receptor agonists.

## Data Availability

The study did not report any data.
